# Supplementary material for: Rapid iceberg calving following removal of tightly packed pro-glacial mélange
Source: Nat Commun. 2019 Jul 19;10:3250. doi: 10.1038/s41467-019-10908-4 (PMC6642183; doi:10.1038/s41467-019-10908-4)
Supplement: Supplementary file 1 — Supplementary Information [file 41467_2019_10908_MOESM1_ESM.pdf]

Supplementary Information for

Rapid iceberg calving following removal of tightly packed pro-glacial mélange

Xie et al.

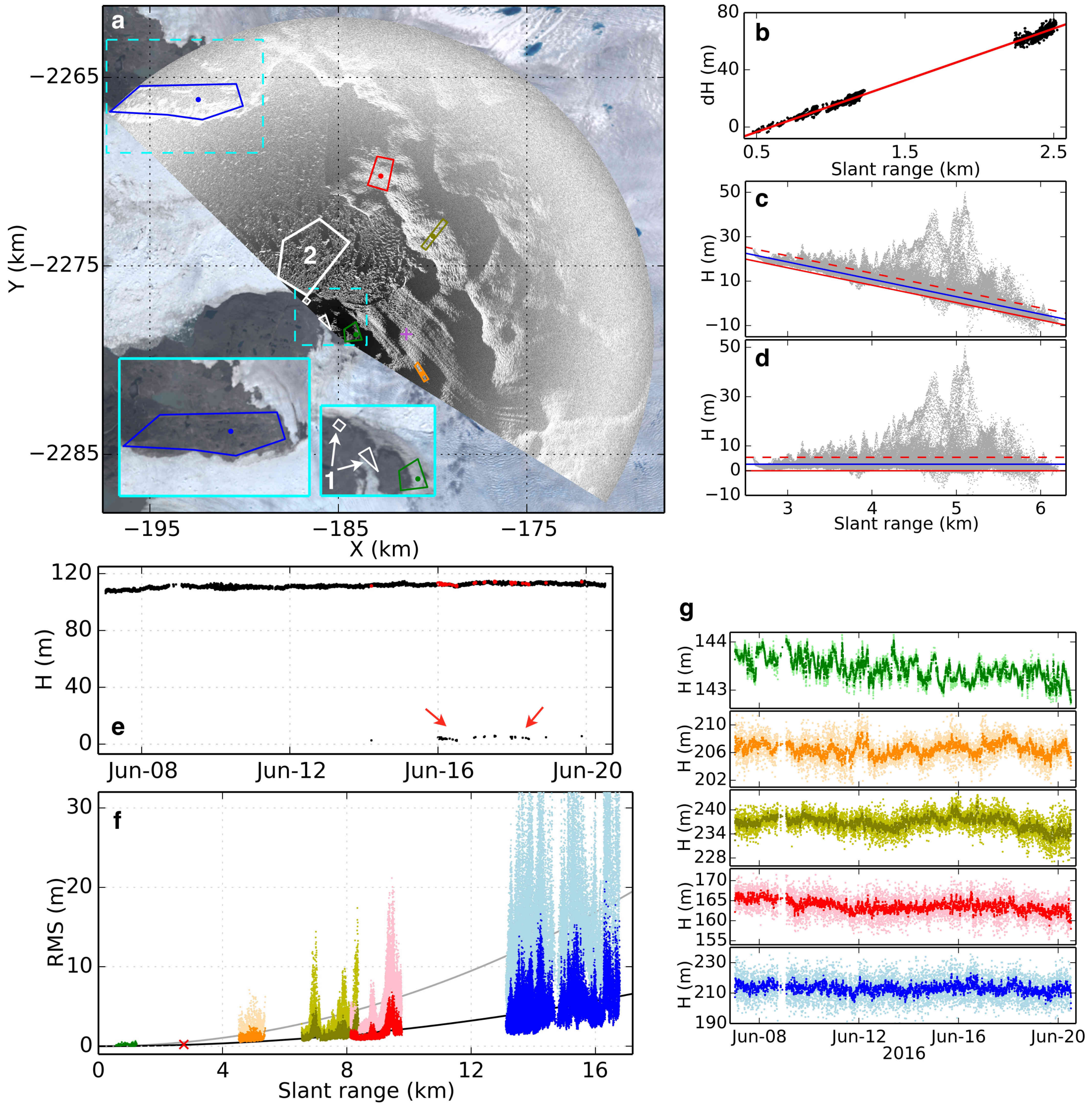

Supplementary Figure 1. Corrections to DEMs and uncertainty estimate. (**a**) shows a TRI amplitude image overlain on a Landsat-8 image. Points within the white triangle and square marked by 1 are used for the first-stage correction as shown in (**b**), where red line is the best fit to height difference between TRI estimate and ArcticDEM. White box in the mélange marked by 2 is the area used to further correct TRI measured heights. Solid and dashed red lines in (**c**, **d**) mark 2 and 80 percentages of measured heights after correction, height at 2 percentage is used to define the mean local sea level, shown by red line. Arrows in (**e**) point to elevations due to phase jumps, red dots are corrected heights. Elevations used in (**e**) corresponds to the purple + symbol in (**a**). Light and dark color dots in (**f**) show RMSs of points within selected boxes outlined by corresponding color in (**a**), red X marks RMS of the model residuals shown in Supplementary Figure 3d. Grey and black curves are best fitting curves to RMSs based on non-smoothed (light color) and 30-minute median filtered elevation time series, respectively. (**g**) shows elevation time series of representative points within corresponding boxes shown by the same color in (**a**). Light and dark colors show non-smoothed and 30-minute median filtered elevation time series.

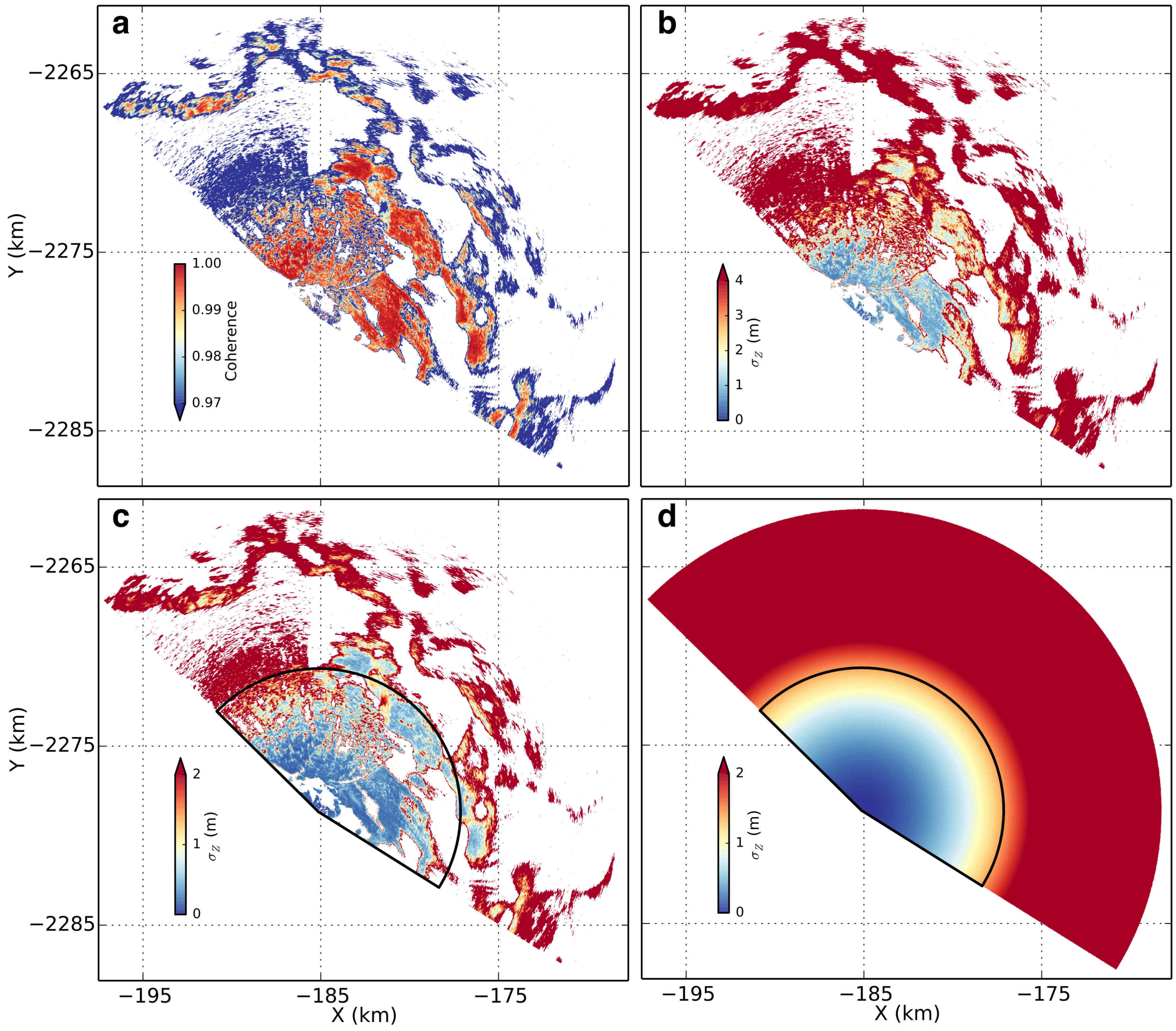

Supplementary Figure 2. Comparison between theoretical uncertainty model based on coherence (a-c) and the model derived from TRI measurements (d). (**a**) shows coherence for a single scan and (**b**) shows the corresponding elevation uncertainty propagated from uncertainty in unwrapped phase based on the model of Rodriguez & Martin[1]. (**c**) shows uncertainty propagated from average of 15 measurements. (**d**) is a predicted uncertainty map by using the uncertainty model derived from TRI measurements (black curve in Supplementary Figure 1f). Black shape in (**c**, **d**) outlines the area within 8 km to the radar, the boxes used to estimate divergence, melt rate, and ice loss of the mélange are all within this area.

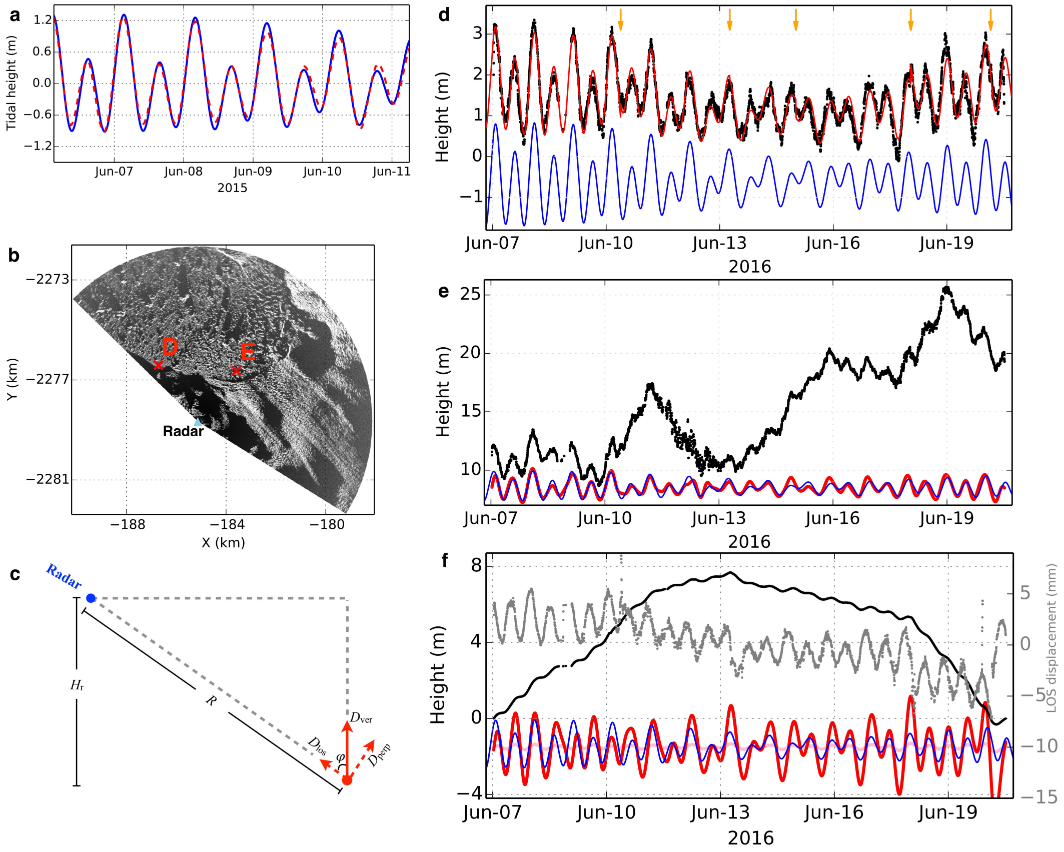

Supplementary Figure 3. Tidally-induced ice elevation changes. (**a**) shows predicted (blue line, based on Richter et al.[2]) and observed (dashed red line, observed from a mooring at the mouth of the fjord) tidal heights in 2015 (the same season), the RMS of differences is 10 cm. (**b**) is a TRI amplitude image showing locations of two points (marked by red X, the same locations as c and f in Fig. 2a of the main paper) used to compare TRI-measured elevation changes with tidal predictions shown in (**d-f**), time series of point D is shown in (**d**), time series of point E (moves approximately perpendicular to radar LOS) is shown in (**e**) and (**f**). (**c**) describes the geometry used to derive tidal heights based on a point with flow direction perpendicular to radar LOS. Blue lines in (**d-f**) show predicted tidal heights, offset for clarity. Black dots in (**d**) and (**e**) show observed elevations. Orange arrows in (**d**) mark major calving-like events. Red curve is the best fitting curve to data, considering tidal heights and non-tidal variations modeled by equation (6) of the main paper, RMS of the residuals is 22 cm. In (**e**), red line are band-pass (0.8<frequency<4 cycle-per-day (cpd) passed) filtered data. (**f**) shows elevation estimates of point E by assuming that observed LOS motion is only caused by vertical motion[3]. Grey dots in (**f**) show observed LOS displacements (motion towards the radar is defined as positive, and motion away from the radar as negative. The scale for the LOS displacement is on the right axis, all other variables using the scale on the left axis). Black dots in (**f**) show the integrated LOS displacement, assuming the initial displacement is 0. Pink color shows a band-pass (0.8-4 cpd passed) filtered time series. Red color show time series by scaling pink dots with a factor of 1/cos(φ), where φ is the angle between radar LOS and vertical direction, $\varphi \approx 86°$ at this location. Pink and red dots and the blue curve are offset by the same constant value for clarity.

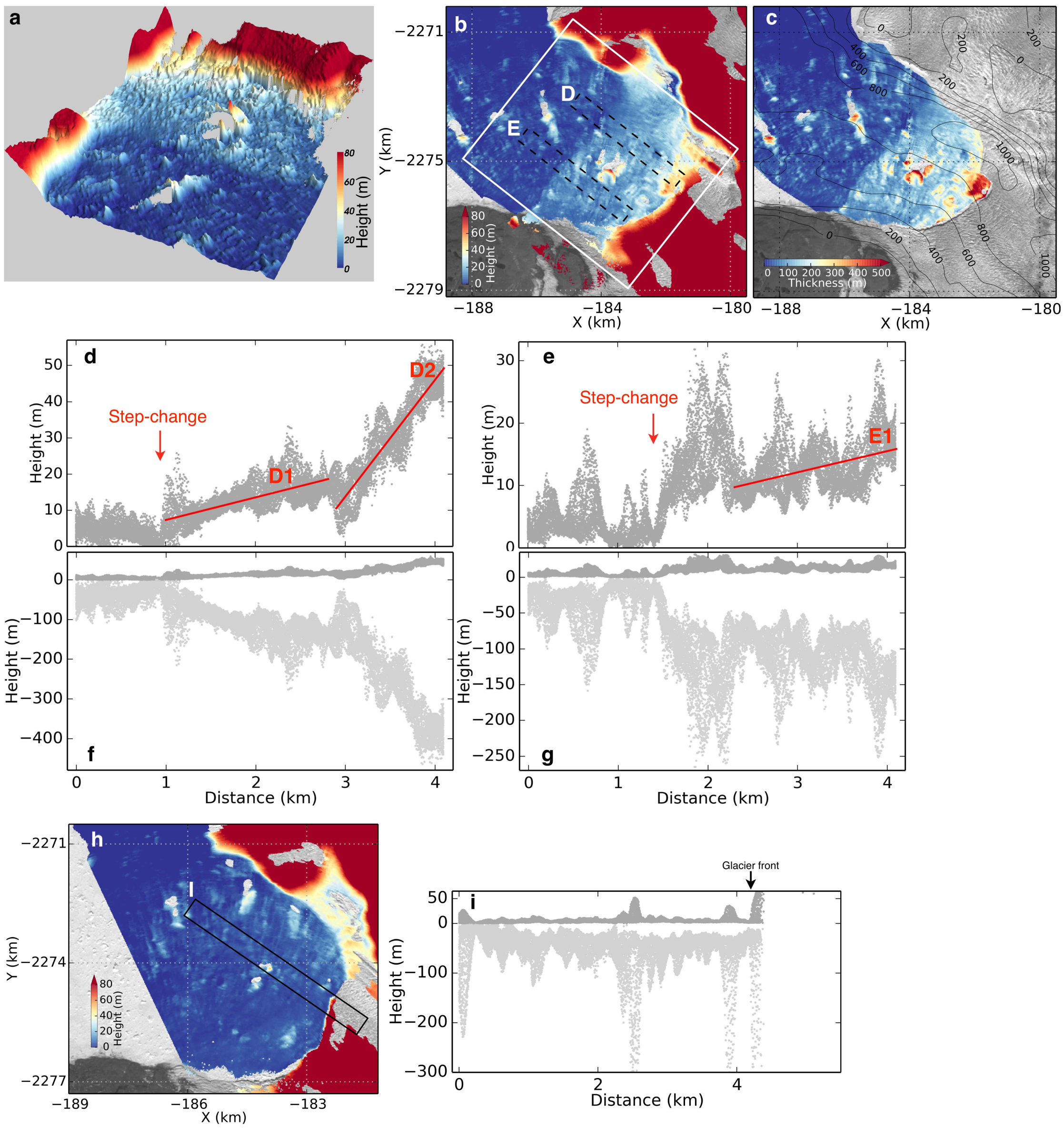

Supplementary Figure 4. Ice surface elevation and thickness of the pro-glacial mélange. (**a**) and (**b**) show a 1 day (13-14 June 2016) median elevation map in 3D and 2D view, (**a**) corresponds to the white box in (**b**), looking from downstream. (**c**) shows mélange thickness assuming ice is in hydrostatic equilibrium, contours show the bed topography[4]. Note that the mélange thickness reaches 200 m for much of the calving front, and the submerged part of the largest iceberg is still smaller than the corresponding bed depth. (**d**) and (**e**) show elevation profiles corresponding to the dashed black boxes D and E in (**b**). Distance is measured from the downstream end. Red arrows in (**d**) and (**e**) mark step-change locations (have lowest elevations due to ice pulled apart). Slopes for D1, D2 and E1 are 0.4°, 1.8° and 0.2°, respectively. Dark and light grey in (**f**) and (**g**) correspond to surface and bottom height. (**i**) shows the outlined profile in (**h**) from a 1 day median of 2015 TRI data[5,6] for comparison, rectangle I in (**h**) has the same location as the rectangle in Fig. 7a of the main paper, color denotes the same as in (**f**) and (**g**).

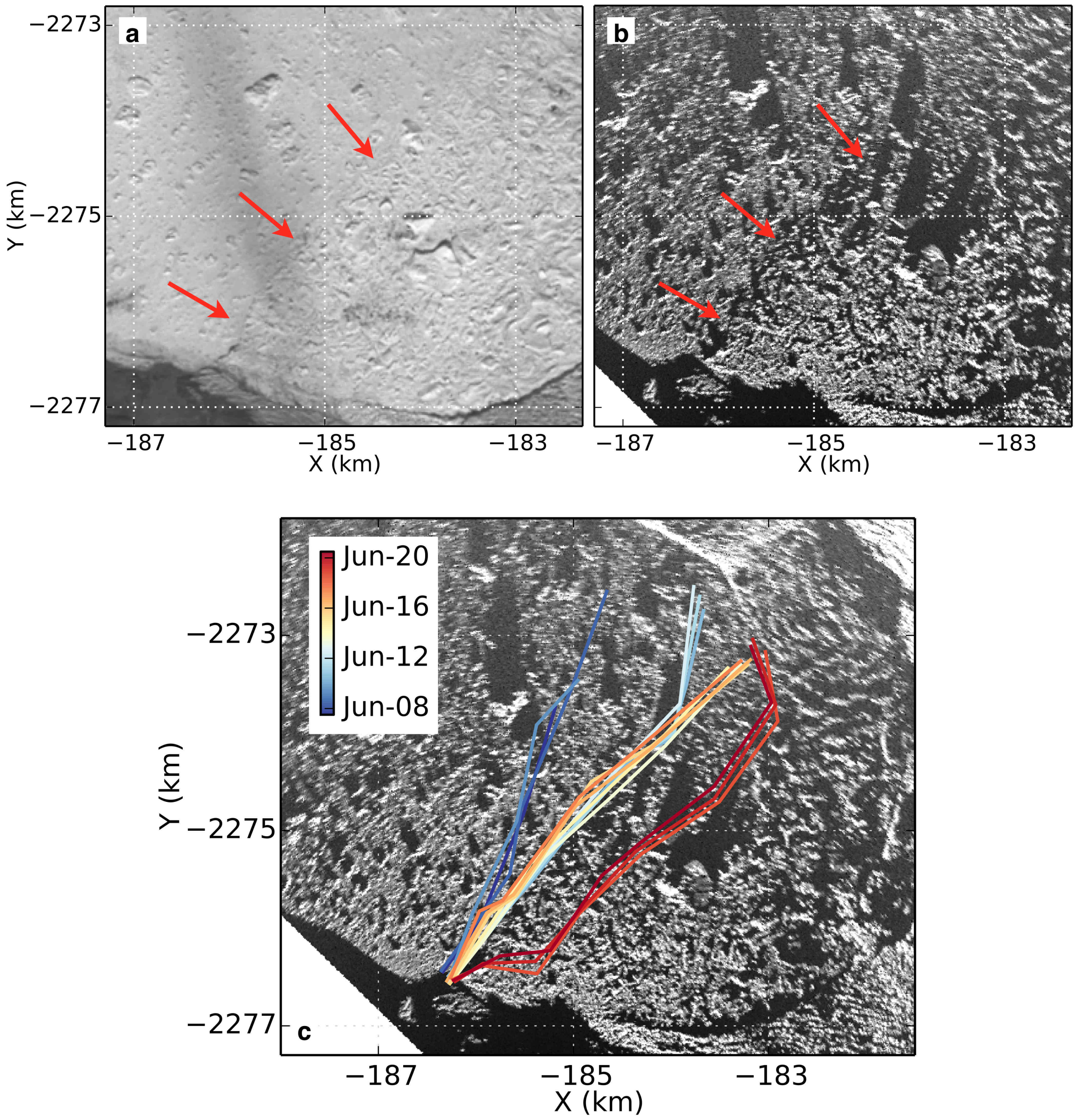

Supplementary Figure 5. Step-change in mélange elevation. Landsat-8 (**a**) and TRI amplitude image (**b**) are both acquired at 13:06 13 June 2016, arrows mark location of the elevation step-change**.** Color lines in (**c**) show elevation step-change at 12:00 each day (inferred from TRI images). Note that upstream mélange is rougher, with numerous large icebergs, while downstream mélange is smoother

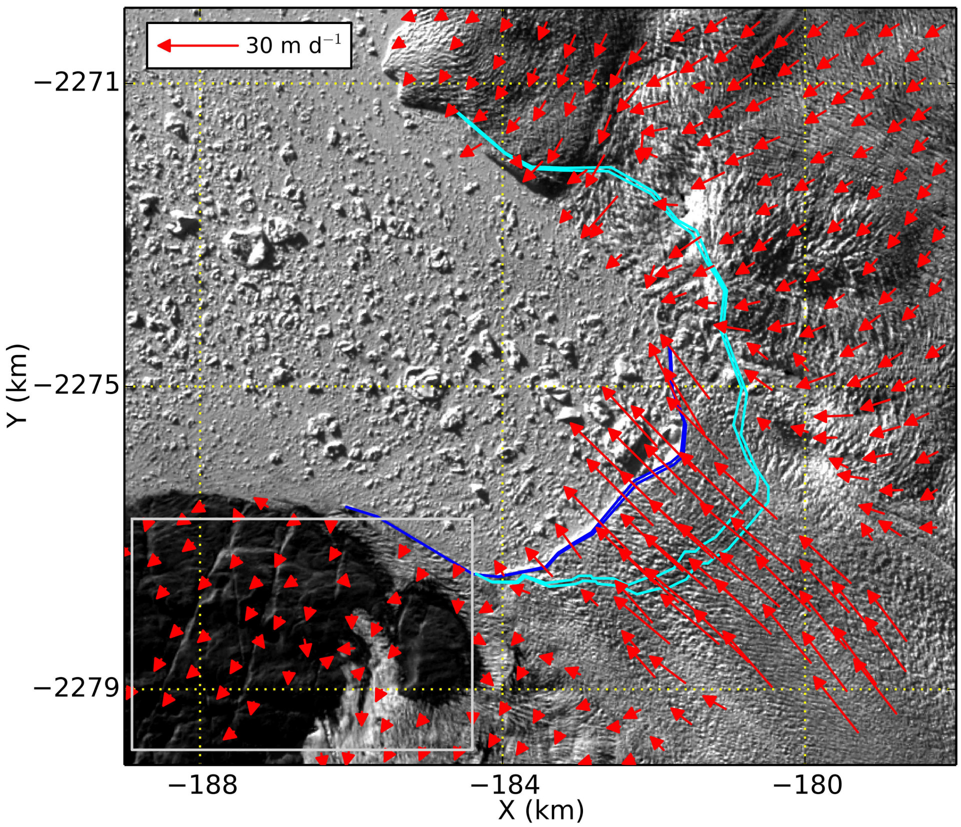

Supplementary Figure 6. Feature tracking of a Landsat-8 image pair. Vectors within the grey box show velocities at (near-) stationary points, we use their RMS as a measure of the uncertainty for feature tracking. Two images were acquired at 00:00 6 June 2016 and 00:00 22 June 2016 (background image). The RMS is 0.29 m d^{-1} and 0.48 m d^{-1} on two orthogonal directions. The blue shape shows ice front motion between 12:20 20 June 2016 and 00:00 22 June 2016 (Figs. 3j-3k of the main paper), the cyan shape shows ice front motion between 00:00 22 June 2016 and 00:06 29 June 2016 (Figs. 3k-3l of the main paper). The difference between these terminus positions is used to estimate the ice loss due to glacier calving.

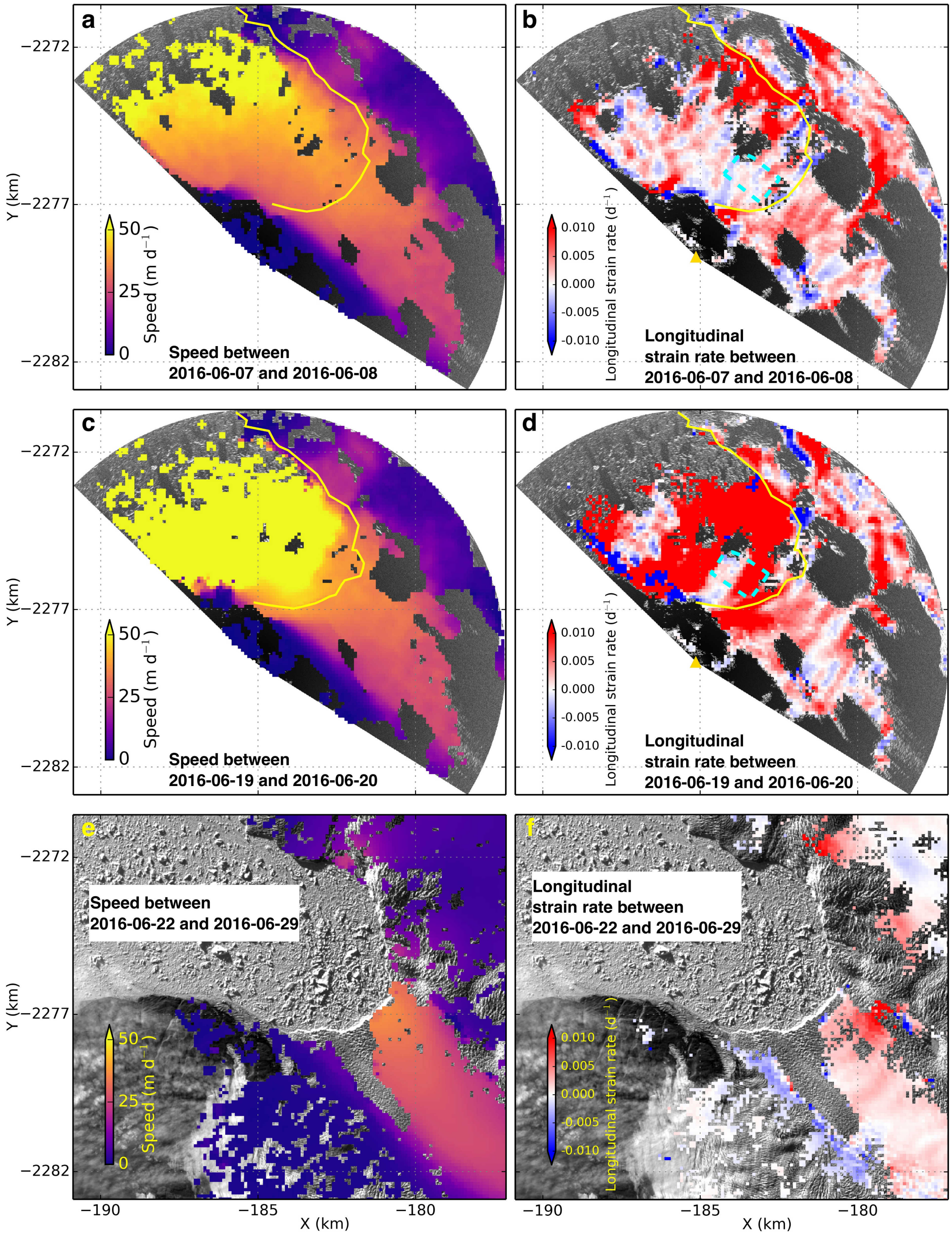

Supplementary Figure 7. Surface speed and longitudinal strain rate. Speeds estimated by feature tracking method, and longitudinal strain rates calculated using the logarithmic strain-rate calculation code of Alley et al.[7], using a 300 m effective length scale. Based on longitudinal and transverse strain rates, we estimated a mean divergence thinning rate of 0.04 m/d within the dashed cyan box of (**b**) on the first TRI observation day, and a mean divergence thinning rate of 1.84 m/d within the dashed cyan box of (**d**) on the last TRI observation day. These are comparable to divergence rate estimates shown in Fig. 5n of the main paper.

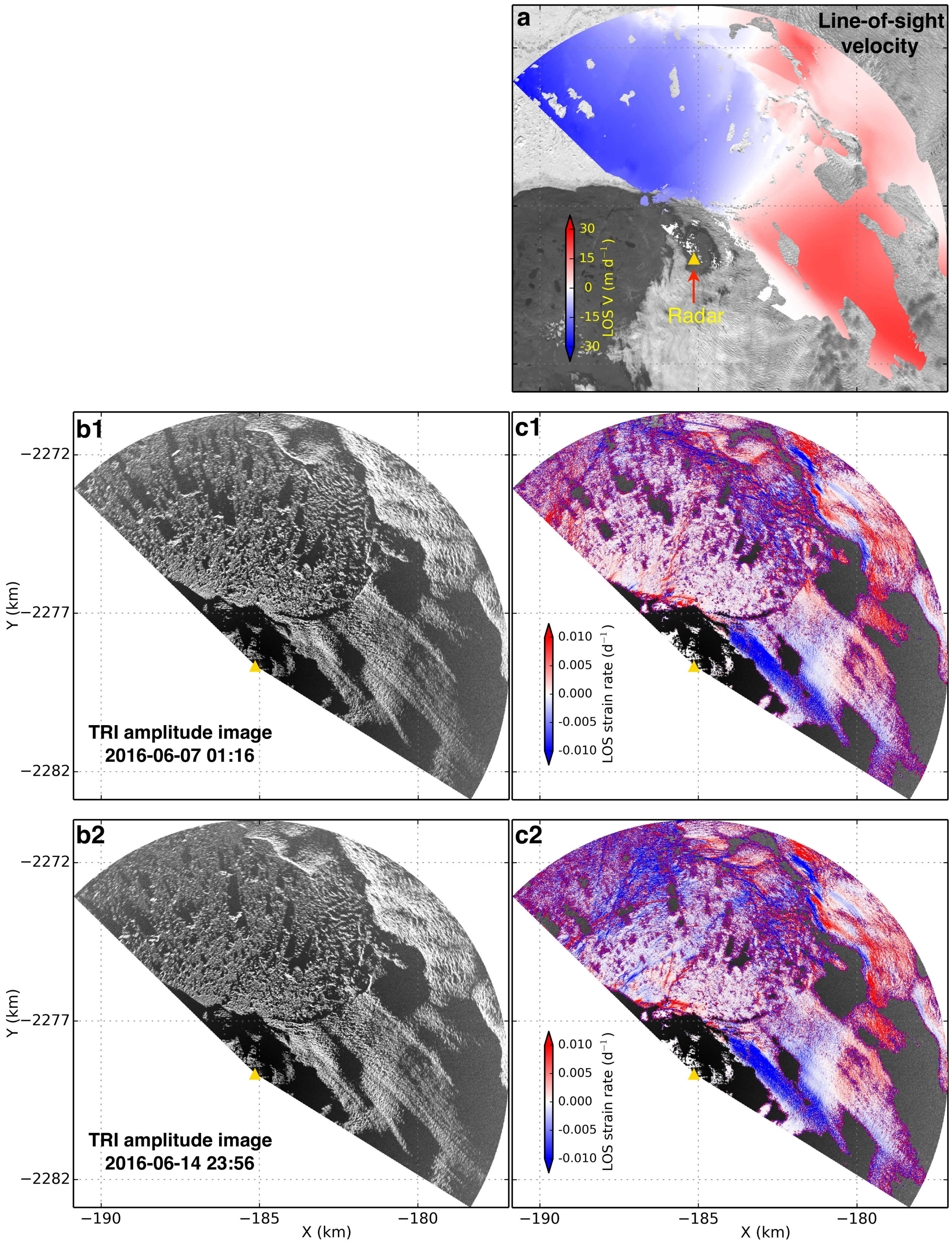

Supplementary Figure 8. Line-of-sight strain rate (continued next page).

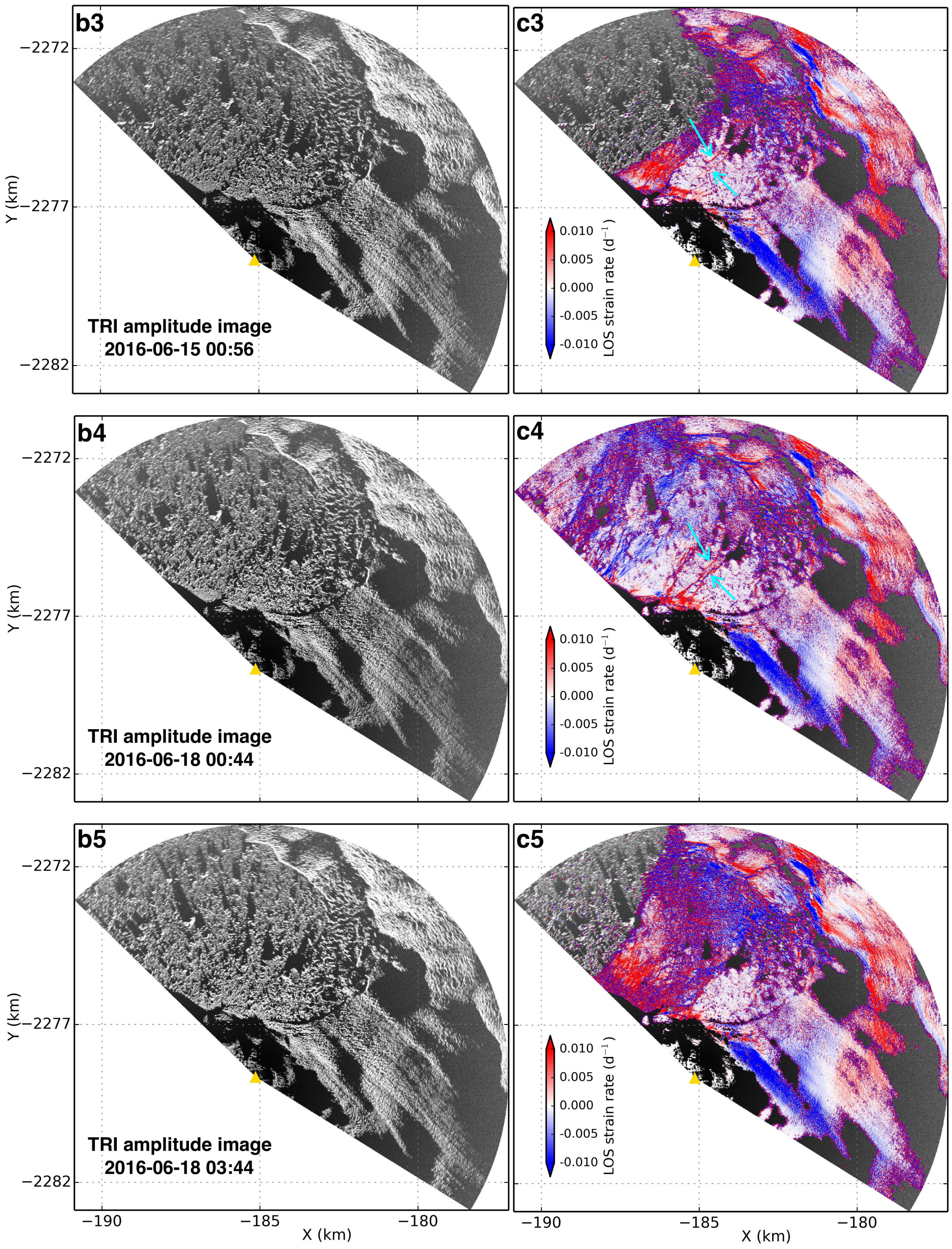

Supplementary Figure 8. Line-of-sight strain rate (continued next page).

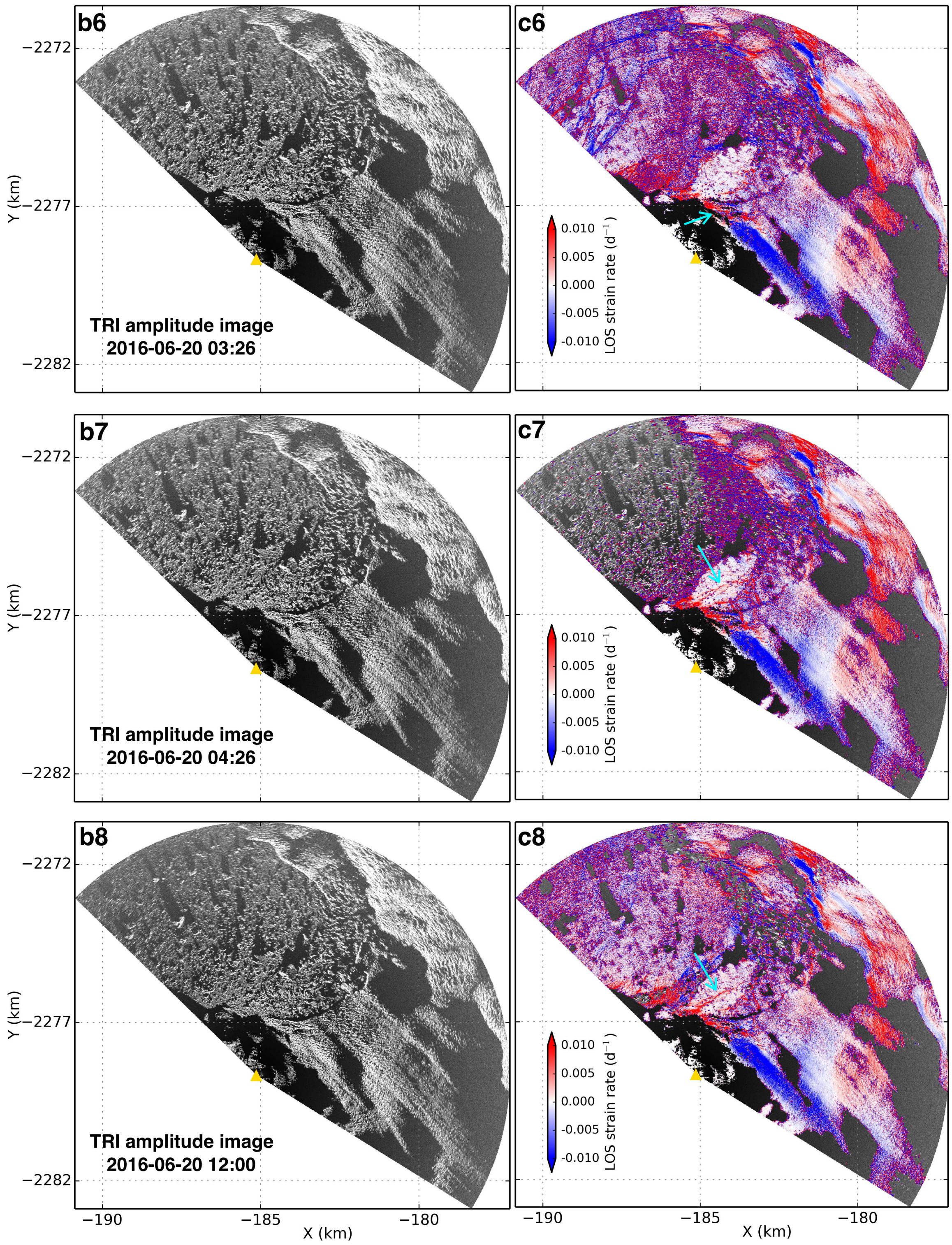

Supplementary Figure 8. Line-of-sight strain rate. (**a**) shows a one-day median average LOS velocity map, adapted from Fig. 8a in Xie et al.[6]. (**b1-b8**) show amplitude images within 8 km to the radar. (**c1-c8**) show LOS strain rates corresponding to the times labeled on the left maps. Arrows mark newly formed fissures after calving-like collapses.

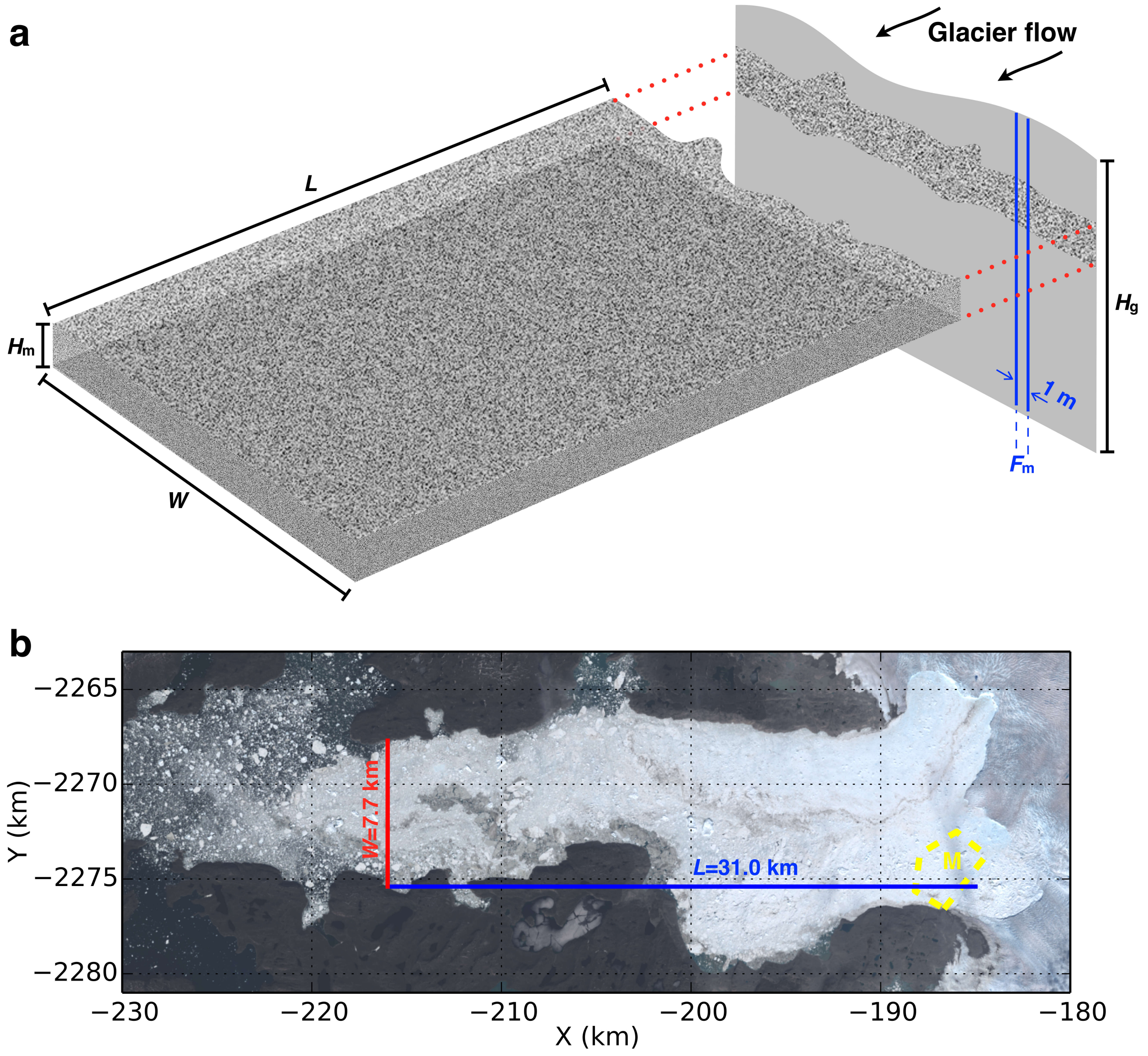

Supplementary Figure 9. **a**, Diagram of the mélange geometry used in buttressing force estimate. Buttressing force in this study is calculated as force per meter lateral width (force acting on the area between the two blue lines). **b**, Dimensions of the mélange estimated from Landsat-8 image. Background is a natural color Landsat-8 image acquired on 13 June 2016. Red line marks simplified width, 7.7 km. Blue line marks simplified length, 31.0 km. Dashed polygon M marks an area downstream from the mélange elevation step-change. Its TRI-derived average thickness is 39.6 m.

Supplementary References

1. Rodriguez E. & Martin J. M. Theory and design of interferometric synthetic aperture radars. *IEE Proc. F (Radar and Signal Processing)* ***139,*** 147-159 (1992).
2. Richter A., Rysgaard S., Dietrich R., Mortensen J. & Petersen D. Coastal tides in West Greenland derived from tide gauge records. *Ocean Dynam.* ***61,*** 39-49 (2011).
3. Voytenko D., Stern A., Holland D. M., Dixon T. H., Christianson K. & Walker R. T. Tidally driven ice speed variation at Helheim Glacier, Greenland, observed with terrestrial radar interferometry, *J. Glaciol.,* ***61,*** 301-308 (2015).
4. An L., Rignot E., Elieff S., Morlighem M., Millan R, Mouginot J., Holland D. M., Holland D. & Paden J. Bed elevation of Jakobshavn Isbræ, West Greenland, from high-resolution airborne gravity and other data, *Geophys. Res. Lett.* ***44,*** 3728-3736 (2017).
5. Xie S., Dixon T. H., Voytenko D., Holland D. M., Holland D. & Zheng T. Precursor motion to iceberg calving at Jakobshavn Isbræ, Greenland, observed with terrestrial radar interferometry. *J. Glaciol.* ***62,*** 1134-1142 (2016).
6. Xie S., Dixon T. H., Voytenko D., Deng F. & Holland D. M. Grounding line migration through the calving season at Jakobshavn Isbræ, Greenland, observed with terrestrial radar interferometry. *The Cryosphere* ***12,*** 1387-1400 (2018).
7. Alley, K.E., Scambos, T.A., Anderson, R.S., Rajaram, H., Pope, A. & Haran, T.M.. Continent-wide estimates of Antarctic strain rates from Landsat 8-derived velocity grids. *Journal of Glaciology*, ***64***(244), 321-332 (2018).
