# Supplementary material for: Rapid iceberg calving following removal of tightly packed pro-glacial mélange
Source: Nat Commun. 2019 Jul 19;10:3250. doi: 10.1038/s41467-019-10908-4 (PMC6642183; doi:10.1038/s41467-019-10908-4)
Supplement: Supplementary file 3 — Description of Additional Supplementary Files [file 41467_2019_10908_MOESM3_ESM.pdf]

Description of Additional Supplementary Files

File Name: Supplementary Movie 1
Description: Major calving-like collapse events in the mélange. Red dot marks the radar location. This movie is composed of selected TRI scans and time runs nonlinearly.

File Name: Supplementary Movie 2
Description: Elevation variation of pro-glacial mélange within 8 km to the radar, view from downstream of the glacier. Elevations are derived from 30-minute median filtered DEMs. Note the step-change of mélange elevation migrates towards the glacier front due to calving-like collapse events.

File Name: Supplementary Movie 3
Description: Ice speed from feature tracking in a daily increment. G1, G2, M1, and M2 correspond to the four points B-E shown in Fig. 8a of the main paper. Light blue triangle shows location of the radar. Dashed white lines mark corresponding LOS directions.
